# miR-325-3p, a novel regulator of osteoclastogenesis in osteolysis of colorectal cancer through targeting S100A4

**DOI:** 10.1186/s10020-021-00282-7

**Published:** 2021-03-10

**Authors:** Li Chengling, Zhang Yulin, Xie Xiaoyu, Lu Xingchen, Zhang Sen, Wang Ziming, Chen Xianming

**Affiliations:** 1 Daping Hospital of Army Medical University, Chongqing, 400042 People’s Republic of China; 2 Chongqing Key Laboratory of Translational Research for Cancer Metastasis and Individualized Treatment, Chongqing University Cancer Hospital and Chongqing Cancer Institute and Chongqing Cancer Hospital, Chongqing, 400030 People’s Republic of China

**Keywords:** miR-325-3p, Bone metastasis, Colorectal cancer cells, S100A4

## Abstract

**Background:**

To investigate effect of microRNA-325-3p (miR-325-3p) on bone metastasis of colorectal cancer (CRC) and the precise role on osteoclastogenesis.

**Methods:**

CT-26 cells were injected into tibias to establish bone metastatic model of CRC in vivo. AgomiR-325-3p or antagomir-325-3p were injected in tail-veins of Balb/c mice to interfere the osteoclastogenesis and bone metastasis of CRC. Safranin O and Fast Green staining examined the changes of trabecular area and TRAP staining examined the osteoclast number in bone metastasis of CRC. Real-time PCR was conducted to test the RNA level of miR-325-3p and mRNA levels of TRAP and Cathepsin K in osteoclast precursors (OCPs). Dual-luciferase reporter system was utilized to identify the direct target of miR-325-3p. Conditioned medium from CT-26 cells was collected to stimulate the OCPs during osteoclastogenesis induced by RANKL and M-CSF in vitro. Western blot analysis was performed to examine the protein level of S100A4 in OCPs after interfered by agomiR-325-3p or antagomir-325-3p cultured in CM or not.

**Results:**

miR-325-3p downregulated in OCPs in CRC microenvironment both in vivo and in vitro. By luciferase activity assay, S100A4 was the target gene of miR-325-3p and the protein level of S100A4 in OCPs upregulated in CRC microenvironment. Overexpression of miR-325-3p inhibited the osteoclastogenesis of OCPs and it can be reversed after transfection with plasmid containing S100A4. Treatment with miR-325-3p can preserve trabecular area in bone metastasis of CRC.

**Conclusion:**

miR-325-3p can prevent osteoclast formation through targeting S100A4 in OCPs. Overexpression of miR-325-3p efficiently decreased the osteoclast number and attenuated bone resorption in bone metastasis of CRC.

## Introduction

Bone is one of metastatic sites in colorectal cancer (CRC). It was reported median time to first skeletal-related event (SRE) was 2 months and median survival in CRC patients was only 7 months since they were diagnosed with bone metastasis, indicating bone metastasis is an early event in CRC and leads to a poorer prognosis (Santini et al. 2012; Qiu et al. 2015). Besides to death, complications, including bone-associated pain, pathological fractures, can influence quality of life (QoL) in patients with CRC significantly. Radiation and pathologic fractures can affect 45% and 10% patients with bone metastasis of CRC, respectively (Santini et al. 2012). Preventing or attenuating the progression of bone metastasis is critical to improve QoL and prognosis of patients with bone metastasis of CRC. However, due to relative lower incidence (Katoh et al. 1995), the underlying mechanisms regulating bone metastasis of CRC are still elusive.

Bone metastasis from CRC presents with osteolytic lesions. Osteoclasts (OC) are one of most important cellular sources that is responsible for progression of bone metastasis of CRC. Aberrant activation of both osteoclastogenesis and osteoclasts contributes to increased number of OCs and worse bone resorption. microRNAs (miRNAs,miRs) are group of small non-coding RNAs, which contains about 22-25nt. The miRNAs can regulate the gene expression at post-transcriptional level. Numerous studies found miRNAs participated in tumor metastasis. Interestingly, both osteoclastogenesis and tumorigenesis of CRC are remarkedly regulated by miRNAs. Previous studies identified miR-483-5p, miR-124, miR-218, miR-199a-5p, miR-133a can enhance the osteoclastogenesis while miR-340 inhibited the osteoclast formation via inhibiting MITF (Li et al. 2018, 2019, 2020; Guo et al. 2018, 2019; Zhao et al. 2017). In addition, miR-16 and miR-378 promoted osteoclastogenesis in bone metastasis of breast cancer (Ell et al. 2013). On the other hand, miRNAs have been identified as efficient prognostic markers of CRC (Motieghader et al. 2017). It was reported that miR-802, miR-99b-5p, miR-20a, miR-499a and miR-576-5p were associated with metastasis of CRC (Zhang et al. 2020; Makondi et al. 2019; Li et al. 2015). Till now, limited studies focused on miRNAs in bone metastasis of CRC, miRNA-141, miRNA-21, miRNA-181a, miRNA-224, miRNA-126 were reported to associate with metastasis and prognosis of CRC (Gao et al. 2018). In addition, miRNA-340 expressing in bone marrow decreased in patients with liver metastasis of CRC (Takeyama et al. 2014). However, it is worthy to find out the miRNAs that directly regulating bone metastasis of CRC which could be potential biomarkers or therapeutical targets.

In this study, we would like to explore the role of miR-325-3p in bone metastasis of CRC both in vitro and in vivo, and to find out the underlying mechanism. Our findings can provide a new miRNA that regulates progression of bone metastasis from CRC.

## Methods

### Animal experiments

All animal experiments and procedures were approved by the Institutional Animal Care and Use Committee at Daping Hospital. BALB/c male mice at 8 to 10 weeks old were used in experiments. The experimental mice were randomly allocated into cages to minimize potential confounders. The experimental mice were randomly allocated to control and treatment groups by using random number table. Mice were housed in a clean room with a 12:12 h light–dark cycle and a temperature of 25 °C. About 8 to 10 mice were kept in one cage and given free access to food and tap water. Before procedures, mice were starved for 12 h. Sample sizes for experiments were determined thorough references containing similar experiments (Ell et al. 2013; Liang et al. 2019; Farr et al. 2017). 50,000 of CT-26 cells, a colorectal cancer cell line, were injected intratibially to establish the bone metastasis of CRC. For miRNA interference experiment, 10 μg agomir-325-3p or antagomir-325-3p were injected into lateral veins weekly.

### Cell isolation

Bone marrow was rinsed out by using PBS. Then the cells were collected and centrifuged at 500*g* for 5 min. The cells were resuspended with FACS buffer and stained with APC-conjugated anti-Cd115 (Biolegend, USA) and PE-conjugated anti-RANK (Biolegend, USA) in a total volume of 100 μL for 30 min on ice in dark. After washing with wash buffer for 3 times, the CD115( +): RANK(−) cells were sorted by FACSAria III (BD Biosciences, USA).

### Cell culture

Primary osteoclast precursors were maintained in Dulbecco’s modified Eagle’s medium (DMEM, Hyclone, USA) containing 10% FBS, 100 μg/mL streptomycin and 100 μg/mL penicillin and treatment with 50 ng/ml colony stimulating factor (M-CSF, Abcam, USA). To induce osteoclast differentiation, cells were exposed to induction medium consisting of RANKL (50 ng/ml) and CSF for 6 days. The 293 T cells were cultured in DMEM containing 10% FSB, 100 μg/mL streptomycin and 100 μg/mL penicillin.

### Indirect co-culture assay

After the CT-26 cells were cultured for 3 days, the medium was discarded and replaced with fresh DMEM and continued to culture for more 24 h. Then the medium was collected to be used as conditioned medium (CM) in later experiments. To test the effect of CM on osteoclastogenesis, the collected CM was added into complete culture medium at 1:1 ratio with/without presence of RANKL and M-CSF to stimulate OCPs.

### TRAP staining assay

For tartrate resistant acid phosphatase (TRAP) stain, the cells or tissue samples were fixed in pre-cooled 4% paraformaldehyde for 15 min and rinsed in deionized water. TRAP staining fluid was added and the samples were incubated at 37℃ for 1 h in dark. After removal of solution, the samples were washed three times. The TRAP positive staining multinuclear cells were recorded using a fluorescence microscope IX81 (Olympus, Japan).

### Histochemistry assay

Tibias were removed from mice at the time of sacrifice and were fixed in 4% paraformaldehyde for 2 days after removing all muscles. The samples were then decalcified in a solution of 10% EDTA for 3 weeks and embedded in paraffin. For Safranin O and Fast Green staining, samples were dewaxed and washed in PBS. Then samples were immersed in 0.1% Safranin O solution for 3 min and washed in PBS for 3 times. Next, samples were immersed in 0.1% Fast Green solution for 10 s and washed in 1% acetum following by washing in PBS for 3 times. After mounting, the sections were observed and captured.

### Transient transfection

miR-325-3p or scramble control and S100A4 expression construct (GenePharma, China) were transfected using Lipofectamine 3000 according to manufacturer’s protocols. After transfected for 48 h, the cells were collected for subsequent experiments.

### Dual-luciferase reporter system assay

293 T cells were seeded into 6-well plates. The cells were transfected with pGL3-S100A4 3′-UTR (Addgene), pRL-TK (Promega, USA) using Lipofectamine RNAiMAX (Invitrogen, USA) following manufacture’s instruction. Luciferase assays were performed with dual-luciferase reporter assay system (Promega, USA) according to the manufacturer’s instructions. The values from the firefly luciferase construct was normalized by Renilla luciferase assay.

### Quantitative real-time PCR

The total RNA was extracted with TRIzol Reagent (Invitrogen, USA). 0.5 μg of RNA was reverse transcribed using RevertAid First Strand cDNA Synthesis kit (Thermo Fisher Scientific, USA) according to manufacturer’s protocols. The cDNA was used for detecting the level of RNA using SYBR Premix Ex TagTMII kit (TakaraBio, Japan). The mRNA levels were normalized to GAPDH and the miRNA level was normalized to U6. The primer sequences was as below: GAPDH (forward: 5′-TGGATTTGGACGCATTGGTC-3′ and reverse: 5′-TTTGCACTGGTACGTGTTGAT-3′), TRAP (forward: 5′-TCACCCTGACCTATGGTGC-3′ and reverse: 5′-GCCGGACTCCAATGTTAAAGC-3′), Cathepsin K (forward: 5′-CTGGCTGGGGTTATGTCTCAA-3′ and reverse: 5′-GGCTACGTCCTTACACACGAG-3′).

### Western blot

Cell extracts was subjected to SDS–PAGE gels and transferred to polyvinylidene fluori (PVDF) membranes. The membrane was blocked with 5% BSA diluted in PBS and then incubated with primary antibody overnight at 4 °C. The blots were then incubated with secondary antibodies labeled with HRP. Signal was detected using a scanner (ChemiDoc Touch Imaging System, USA). The primary and secondary antibodies used were as below: Rabbit anti-mouse S100A4 antibody (Cell Signaling Technology, USA), Rabbit anti-mouse GAPDH (Cell Signaling Technology, USA) and HRP-conjugated IgGs (Cell Signaling Technology, USA).

### Statistical analysis

Results are showed as means ± standard error (SE) or standard deviation (SD) as required. Independent samples were used in all experiments. Student's *t*-test was used in comparison of two groups. For comparing three or above groups, data were analyzed by analysis of variance with one way ANOVA followed by Dunnett’s post hoc tests. ANOVA assumptions were checked by using Kolmogorov–Smirnov test. GraphPad Prism version 8.0 (San Diego, CA) was used for analyzing data. Statistical significance was considered at p < 0.05.

## Results

### miR-325-3p downregulates in osteoclast precursors in bone metastasis of CRC

**Fig. 1:**
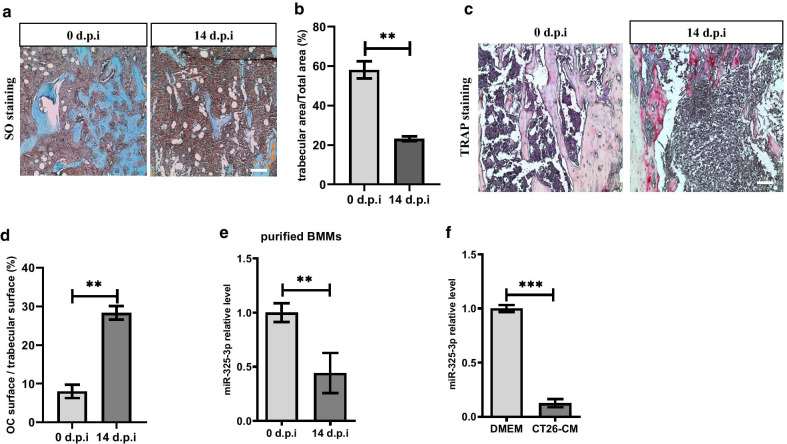
miR-325-3p downregulates in osteoclast precursors in bone metastasis of CRC. **a** 100,000 CT-26 cells were injected into tibias in mice and the samples were collected at specific timepoints to be sectioned. Safranin O-Fast Green staining revealed trabecular area (Scar bar = 50 μm) (n = 3 mice). **b** Quantification of trabecular area in (a) (mean ± SE). **c** TRAP staining revealed TRAP positive cells in trabecular area at specific timepoints after injection of CT-26 cells (Scar bar = 50 μm) (n = 3 mice). **d** Quantification of relative area of osteoclast surface in (**c**) (mean ± SE). **e** Primary OCPs were isolated from bone marrow at specific timepoints through FACS. Real-time PCR analysis showed the relative expression of miR-325-3p in OCPs at 14 d.p.i comparing with OCPs at 0 d.p.i. (D0, n = 3 mice; D14, n = 5 mice) (mean ± SD). **f.** Primary OCPs were isolated and cultured in DMEM or CT-26 CM for 4 days in the presence of RANKL and M-CSF. Real-time PCR analysis showed the relative expression of miR-325-3p in OCPs treated by CM or DMEM (mean ± SD). In vitro experiments were performed at least 3 times. *p < 0.05, **p < 0.01, ***p < 0.001

Safranin O and Fast Green staining showed comparing with samples collected at 0 days post injection (0 d.p.i), trabecular area significantly decreased in samples collected at 14 d.p.i (Fig. 1a, b). Consistent with these changes, TRAP staining revealed the area of TRAP ( +) cells in samples at 14 d.p.i increased significantly comparing with samples at 0 d.p.i (Fig. 1c, d). These findings indicated CT-26 cells efficiently promoted the osteolysis in tibial and leaded to aberrant activation of osteoclasts.

CD115 ( +) RANK (−) precursors (thereafter named osteoclast precursors, OCPs) derived from bone marrow were identified as primary early osteoclast precursors (Farr et al. 2017). To examine the level change of miR-325-3p, we sorted out OCPs from affected tibias at 0 d.p.i and 14 d.p.i via FACS, respectively. Real-time PCR analysis showed the level of miR-325-3p remarkedly decreased at 14 d.p.i (Fig. 1e). To investigate whether CT-26 cells downregulate the expression of miR-325-3p in OCPs, conditioned medium (CM) collected from CT-26 cells were used to stimulate OCPs, the result showed the expression of miR-325-3p also downregulated in CM treated OCPs (Fig. 1f). These findings indicated that CT-26 cells promoted the osteolytic lesion formation and the expression of miR-325-3p was negatively associated with the severity of bone destruction and the number of osteoclasts.

### miR-325-3p regulates CRC stimulated osteoclastogenesis

**Fig. 2:**
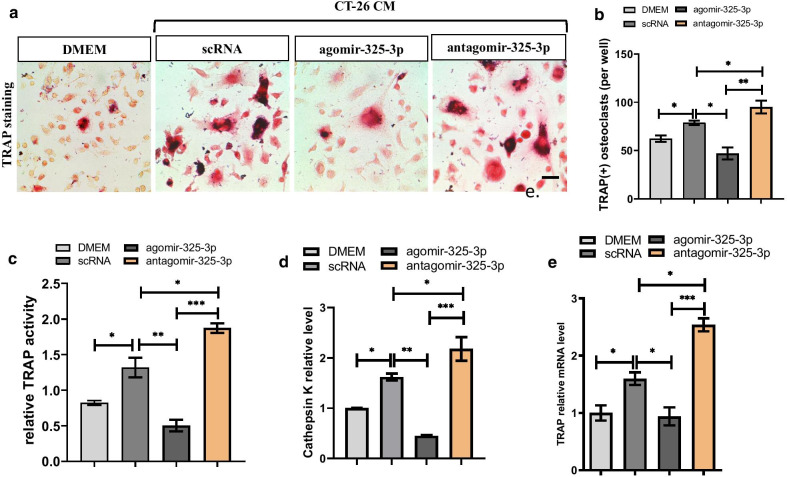
miR-325-3p regulates CRC stimulated osteoclastogenesis. **a** OCPs were isolated through FACS and maintained in DEME or CT-26 CM and transfected with agomir-325-3p, antagomir-325-3p or negative controls in the presence of RANKL and M-CSF for 4 days. TRAP positive multinucleated cells with more than three nuclei were counted as mature osteoclasts (Scar bar = 10 μm). **b** Quantification of osteoclast number in each group in (**a**) (mean ± SE). **c** Relative TRAP activity assay indicated the TRAP activity changes in each group in (**a**) (mean ± SE). **d** Real-time PCR analysis showed the mRNA levels of Cathepsin K in each group in (**a**) (mean ± SD). **e** Real-time PCR analysis showed the mRNA level of TRAP in each group in (**a**) (mean ± SD). In vitro experiments were performed at least 3 times. *p < 0.05, **p < 0.01, ***p < 0.001

Osteolytic tumor cells can promote osteoclastogenesis through secreting osteolytic cytokines in RANKL dependent and independent ways. To verify the effect of miR-325-3p on osteoclastogenesis stimulated by CRC cells, we added CM into culture medium of osteoclast precursors at ratio of 1:1 with/without miR-325-3p or its inhibitor in the presence of RANKL and M-CSF. The results showed secreta from CT-26 cells can significantly enhance the osteoclastogenesis of OCPs, the number of osteoclasts, the activity of TRAP and the mRNA levels of Cathepsin K and TRAP, two important osteoclastogenic markers, all upregulated in OCPs treated by CM comparing with control in the presence of RANKL after stimulating 6 days (Fig. 2a–e). Then we investigated whether miR-325-3p influence the osteoclastogenesis stimulated by secreta from CT-26 cells. After transfection with agomir-325-3p or antagomir-325-3p, the number of osteoclasts decreased or increased comparing with control group, respectively (Fig. 2a, b). In addition, Relative TRAP activity assay also revealed TRAP activity in OCPs can be decreased after overexpression of miR-325-3p and increased after inhibiting the expression of miR-325-3p (Fig. 2c). Meanwhile, the mRNA levels of Cathepsin K and TRAP were downregulated in miR-325-3p overexpressed group and upregulated in miR-325-3p inhibited group (Fig. 2d, e), Together, these results indicated miR-325-3p can inhibit the enhanced osteoclastogenesis caused by secreta of CRC cells.

### MiR-325-3p directly targets S100A4

**Fig. 3:**
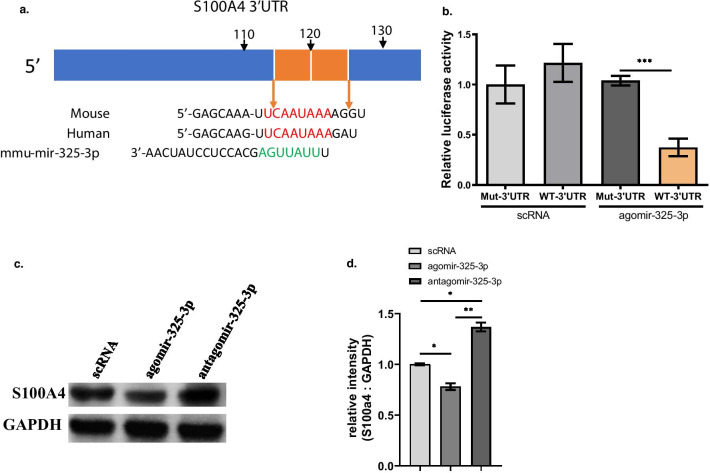
MiR-325-3p directly targets S100A4. **a** The complementary sequences of miR-325-3p were identified in 3′UTR of S100A4 mRNA using TargetScan. **b** miR-325-3p inversely modulated the luciferase activity of plasmids containing WT 3′UTR of S100A4 (WT, Wild Type; Mut, Mutant Type) (mean ± SD). **c** The protein level of S100A4 in OCPs transfected with agomir-325-3p, antagomir-325-3p or their controls were tested by Western blot. **d** The relative intensity of S100A4 comparing with GAPDH in (**c**) was quantified (mean ± SE). In vitro experiments were performed at least 3 times. *p < 0.05, **p < 0.01, ***p < 0.001

To explore the underlying mechanism that miR-325-3p prevents the ostoeclastogenesis of OCPs, we predicted the potential target genes of miR-325-3p at TargetScan. The predicting results showed the region between 117 and 124 of S100A4 3′UTR could be the binding site of miR-325-3p and this sequence is highly conserved between mouse and human (Fig. 3a). Notably, the luciferase activity of 293 T cells that were transfected with wild type (wt) 3′UTR of S100A4 was reduced by miR-325-3p while the luciferase activity in group transfected with mutated type (mut) 3′UTR of S100A4 was not affected. In addition, the miRNA control also had no effects on luciferase activity in both wt group and mut group (Fig. 3b), These results demonstrated that miR-325-3p can bind to 3′ UTR of S100A4 specifically. Then we further investigated the effect of miR-325-3p on protein expression of S100A4 in OPCs. Western blot analysis showed agomiR-325-3p enhanced the protein expression of S100A4 and antagomiR-325-3p inhibited the protein expression (Fig. 3c, d). These findings together indicated S100A4 is target gene of miR-325-3p.

### S100A4 is needed for miR-325-3p mediated downregulation of osteoclast formation in CRC microenvironment

**Fig. 4:**
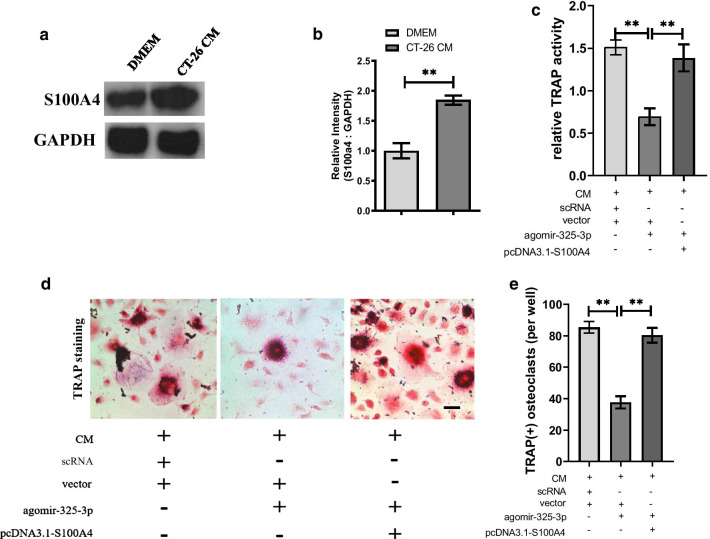
S100A4 is needed for miR-325-3p mediated downregulation of osteoclast formation in CRC microenvironment. **a** OCPs were treated by DMEM or CT-26 CM for 4 days in the presence of RANKL and M-CSF. The protein level of S100A4 in OCPs were analyzed by using Western blot assay. **b** The relative intensity of S100A4 comparing with GAPDH in (**a**) was quantified (mean ± SD). **c** OCPs were transfected with agomir-325-3p or agomir-325-3p with plasmid containing S100A4 and cultured in CM for 4 days in the presence of RANKL and M-CSF. Relative TRAP activity in each group was analyzed (mean ± SE). **d** Representative images for osteoclastogenesis of OCPs cultured in CM after transfection with agomir-325-3p or agomir-325-3p with plasmid containing S100A4 in the presence of RANKL and M-CSF (Scar bar = 10 μm). TRAP positive multinucleated cells with more than three nuclei in each group were counted as mature osteoclasts. **e** The number of osteoclasts in (**d**) was qualified (mean ± SD). In vitro experiments were performed at least 3 times. *p < 0.05, **p < 0.01, ***p < 0.001

Then we tested whether the protein level of S100A4 changed in CRC microenvironment. Western blot analysis revealed the protein level of S100A4 can be upregulated significantly in OCPs after treated with CM comparing with control group (Fig. 4a, b), indicating secreta from CRC cells can upregulate the expression of S100A4. S100A4 was reported to regulate the osteoclastogenesis, thus we next investigated whether the effect of miR-325-3p on osteoclastogenesis in CRC microenvironment was through targeting S100A4. During osteoclastogenesis of OCPs stimulated by CM and RANKL, the number of TRAP ( +) osteoclasts decreased after transfection with miR-325-3p, however, this negative effect was reversed after transfected plasmid containing S100A4 (Fig. 4c–e). These findings indicate that miR-325-3p inhibits osteoclastogenesis of OPCs through targeting S100A4.

### MiR-325-3p treatment attenuates bone resorption in bone metastasis of CRC

**Fig. 5:**
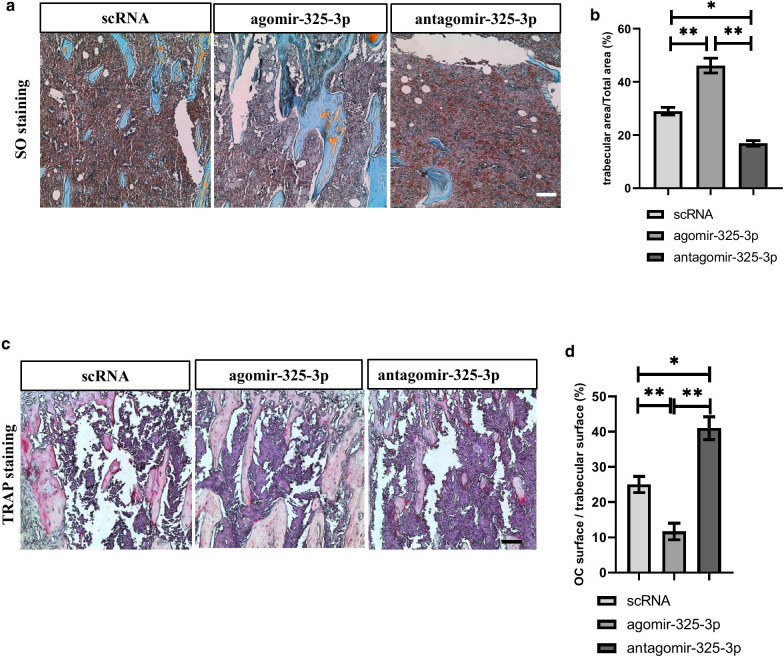
MiR-325-3p treatment attenuates bone resorption in bone metastasis of CRC. **a** The agomir-325-3p or antagomir-325-3p or their controls were injected through lateral veins weekly for three weeks after injection of CT-26 cells intratibially. The samples were collected and sectioned. Safranin O-Fast Green staining revealed trabecular area in each group (Scar bar = 50 μm) (n = 3–5 mice in each group). **b** Quantification of trabecular area in (**a**) (mean ± SE). **c** TRAP staining revealed TRAP positive cells in trabecular area (Scar bar = 50 μm) (n = 3–5 mice in each group). **d** Quantification of osteoclast surface comparing with trabecular surface in (**c**) (mean ± SE). In vitro experiments were performed at least 3 times.*p < 0.05, **p < 0.01, ***p < 0.001

Considering the inhibiting effect of miR-325-3p on osteoclastogenesis, we next investigated the potential therapeutical effects of miR-325-3p on bone metastasis of CRC in vivo. 10 μg agomiR-325-3p or antagomiR-325-3p was injected into lateral vein weekly for three weeks after establishing bone metastatic model of CRC. After 3 weeks, histochemistry analysis showed trabecular area significantly increased in agomiR-325-3p treated group and decreased remarked in antagomiR-325-3p treated group. The percentage of trabecular in total area in agomir-325-3p treated group increased about 1.5 folds comparing to control group. On the contrary, after treatment with antagomir-325-3p, the trabecular area decreased about 10% comparing to control group, indicating miR-325-3p can restore the bone volume (Fig. 5a, b). TRAP staining indicated that relative area of TRAP ( +) osteoclasts comparing with trabecular surface decreased after treated with agomiR-325-3p and increased after treated with antagomiR-325-3p (Fig. 5c, d). Consistent with the findings in vitro, the in vivo experiments indicated that miR-325-3p can prevent the activation of osteoclasts and thus attenuate the bone resorption during bone metastasis of CRC.

## Discussion

In this study, we identified a novel miRNA, miR-325-3p, can regulate the osteoclastogenesis in CRC environment through targeting S100A4. miR-325-3p was once identified as a tumor suppressor, it can inhibit the proliferation and induce apoptosis of hepatocellular carcer cells through suppressing aquaporin 5 (Zhang et al. 2019) and can inhibit the metastasis of non-small cell lung cancer (NSCLC) cells through targeting KIF2C (Gan et al. 2019), indicating miR-325-3p is closely associated with the occurrence and progression of cancers. In our study, we identified the level of miR-325-3p downregulated in both primary OCPs directly isolated from metastatic bone of CRC and OCPs cultured in CRC conditioned medium. Furthermore, overexpression of miR-325-3p can restore the bone volume during bone metastasis of CRC. Our findings indicated that miR-325-3p can be a potential biomarker for indicating the bone resorption of CRC and a therapeutical candidate. To our best knowledge, limited studies focused on the effect of miR-325-3p on bone remodeling. It was reported that miR-325-3p in cementoblasts can be induced by IL-1β and prevent the cementum regeneration in inflammatory microenvironment through targeting Runx2 (Wang et al. 2020), indicating miR-325-3p may have a “double-edged sword” effect on bone remodeling. In microenvironment of CRC, bone resorption was predominant, and the abnormal activation of osteoclasts dominantly regulate this process, overexpression of miR-325-3p in osteoclast precursors can prevent the osteolytic changes and restore the bone volume.

S100A4 is a member of S100 calcium binding protein family. It was identified to be closely associated with tumor metastasis, transfection of S100A4 can enhance the tumorigenic potential and stimulate the metastasis in vivo (Ebralidze et al. 1989; Davies et al. 1993). Afterwards, more evidence indicated S100A4 broadly participates in tumor progression and metastasis in varieties of cancers, especially in CRC. It was found S100A4 could be a promising candidate biomarker for prediction of CRC (Fei et al. 2017; Saleem et al. 2005; Gongoll et al. 2002). The expression of S100A4 in CRC cells is associated with epithelial-to-mesenchymal transition (EMT) and promotes the CRC metastasis phenotype through modulating TGF-β signaling pathway (Wang et al. 2014). In addition, nuclear expression of S100A4 is associated with cancer metastasis of CRC patients (Boye et al. 2010). Interestingly, several studies demonstrated that induction of S100A4 promote the osteoclast formation and bone destruction of osteolytic tumors (Mah et al. 2015; Kim et al. 2019; Erlandsson et al. 2013). Consistently with these findings, we explored that S100A4 upregulated significantly in OCPs during CRC microenvironment and overexpression of S100A4 can promote the CRC-induced osteoclastogenesis. The expression of S100A4 can be modified in transcriptional level and post-transcriptional level. It was reported that LASP1 and Wnt/β-catenin/TCF signaling are two upstream of S100A4 (Stein et al. 2006). miR-296, miR-520c were identified to regulate the metastasis of CRC through modulating S100A4 (He et al. 2017; Mudduluru et al. 2017). In this study, we found miR-325-3p could be a new epigenetic modulator of S100A4. We revealed the binding site of miR-325-3p in S100A4 3′UTR region and proved that the protein level of S100A4 can be efficiently inhibited by miR-325-3p. Moreover, overexpression of miR-325-3p can efficiently downregulate the protein level of S100A4 in OCPs and prevented S100A4-induced osteoclastogenesis in CRC microenvironment.

## Conclusion

**Fig. 6:**
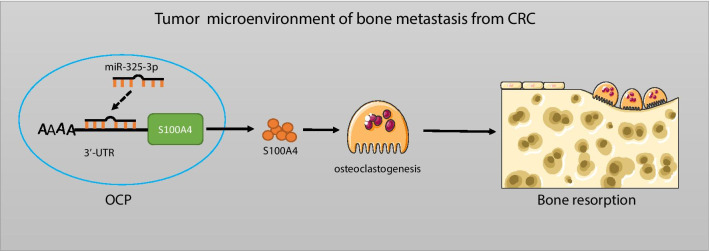
Schematic of the underlying mechanism of miR-325-3p regulating osteoclastogenesis in metastatic bone of CRC

Taken together, we identified the expression of miR-325-3p and S100A4 was downregulated and upregulated in CRC, respectively. Furthermore, miR-325-3p is a key regulator of S100A4 in OCPs in bone metastasis of CRC. The imbalance between miR-325-3p and S100A4 promotes the osteoclastogenesis and regulates the bone resorption in CRC microenvironment (Fig. 6). In addition, miR-325-3p treatment is a promising therapeutical target for preventing CRC associating bone destruction. However, the effect of miR-325-3p on bone growth was not investigated, it is interesting to explore whether its negative effect on osteoclastogenesis can promote the progression of diseases featuring with osteosclerosis in further investigations.

## Data Availability

The datasets during and/or analysed during the current study available from the corresponding author on reasonable request.

## References

[CR1] Boye K, Nesland JM, Sandstad B (2010). Nuclear S100A4 is a novel prognostic marker in colorectal cancer. Eur J Cancer.

[CR2] Davies BR, Davies MP, Gibbs FE (1993). Induction of the metastatic phenotype by transfection of a benign rat mammary epithelial cell line with the gene for p9Ka, a rat calcium-binding protein, but not with the oncogene EJ-ras-1. Oncogene.

[CR3] Ebralidze A, Tulchinsky E, Grigorian M (1989). Isolation and characterization of a gene specifically expressed in different metastatic cells and whose deduced gene product has a high degree of homology to a Ca2+-binding protein family. Genes Dev.

[CR4] Ell B, Mercatali L, Ibrahim T (2013). Tumor-induced osteoclast miRNA changes as regulators and biomarkers of osteolytic bone metastasis. Cancer Cell.

[CR5] Erlandsson MC, Svensson MD, Jonsson IM (2013). Expression of metastasin S100A4 is essential for bone resorption and regulates osteoclast function. Biochim Biophys Acta.

[CR6] Farr JN, Xu M, Weivoda MM (2017). Targeting cellular senescence prevents age-related bone loss in mice. Nat Med.

[CR7] Fei F, Qu J, Zhang M (2017). S100A4 in cancer progression and metastasis: a systematic review. Oncotarget.

[CR8] Gan H, Lin L, Hu N (2019). KIF2C exerts an oncogenic role in nonsmall cell lung cancer and is negatively regulated by miR-325-3p. Cell Biochem Funct.

[CR9] Gao S, Zhao ZY, Wu R (2018). Prognostic value of microRNAs in colorectal cancer: a meta-analysis. Cancer Manag Res.

[CR10] Gongoll S, Peters G, Mengel M (2002). Prognostic significance of calcium-binding protein S100A4 in colorectal cancer. Gastroenterology.

[CR11] Guo K, Zhang D, Wu H (2018). MiRNA-199a-5p positively regulated RANKL-induced osteoclast differentiation by target Mafb protein. J Cell Biochem.

[CR12] Guo J, Zeng X, Miao J (2019). MiRNA-218 regulates osteoclast differentiation and inflammation response in periodontitis rats through Mmp9. Cell Microbiol.

[CR13] He Z, Yu L, Luo S (2017). miR-296 inhibits the metastasis and epithelial-mesenchymal transition of colorectal cancer by targeting S100A4. BMC Cancer.

[CR14] Katoh M, Unakami M, Hara M (1995). Bone metastasis from colorectal cancer in autopsy cases. J Gastroenterol.

[CR15] Kim H, Kim B, Il Kim S (2019). S100A4 released from highly bone-metastatic breast cancer cells plays a critical role in osteolysis. Bone Res.

[CR16] Li W, Chang J, Wang S (2015). miRNA-99b-5p suppresses liver metastasis of colorectal cancer by down-regulating mTOR. Oncotarget.

[CR17] Li Z, Zhang W, Huang Y (2018). MiRNA-133a is involved in the regulation of postmenopausal osteoporosis through promoting osteoclast differentiation. Acta Biochim Biophys Sin (Shanghai).

[CR18] Li B, Wu P, Fu W (2019). The role and mechanism of miRNA-1224 in the polygonatum sibiricum polysaccharide regulation of bone marrow-derived macrophages to osteoclast differentiation. Rejuvenation Res.

[CR19] Li K, Chen S, Cai P (2020). MiRNA-483-5p is involved in the pathogenesis of osteoporosis by promoting osteoclast differentiation. Mol Cell Probes.

[CR20] Liang M, Ma Q, Ding N (2019). IL-11 is essential in promoting osteolysis in breast cancer bone metastasis via RANKL-independent activation of osteoclastogenesis. Cell Death Dis.

[CR21] Mah SJ, Lee J, Kim H (2015). Induction of S100A4 in periodontal ligament cells enhances osteoclast formation. Arch Oral Biol.

[CR22] Makondi PT, Wei PL, Huang CY (2019). Development of novel predictive miRNA/target gene pathways for colorectal cancer distance metastasis to the liver using a bioinformatic approach. PLoS ONE.

[CR23] Motieghader H, Kouhsar M, Najafi A (2017). mRNA-miRNA bipartite network reconstruction to predict prognostic module biomarkers in colorectal cancer stage differentiation. Mol Biosyst.

[CR24] Mudduluru G, Ilm K, Fuchs S (2017). Epigenetic silencing of miR-520c leads to induced S100A4 expression and its mediated colorectal cancer progression. Oncotarget.

[CR25] Qiu M, Hu J, Yang D (2015). Pattern of distant metastases in colorectal cancer: a SEER based study. Oncotarget.

[CR26] Saleem M, Adhami VM, Ahmad N (2005). Prognostic significance of metastasis-associated protein S100A4 (Mts1) in prostate cancer progression and chemoprevention regimens in an autochthonous mouse model. Clin Cancer Res.

[CR27] Santini D, Tampellini M, Vincenzi B (2012). Natural history of bone metastasis in colorectal cancer: final results of a large Italian bone metastases study. Ann Oncol.

[CR28] Stein U, Arlt F, Walther W (2006). The metastasis-associated gene S100A4 is a novel target of beta-catenin/T-cell factor signaling in colon cancer. Gastroenterology.

[CR29] Takeyama H, Yamamoto H, Yamashita S (2014). Decreased miR-340 expression in bone marrow is associated with liver metastasis of colorectal cancer. Mol Cancer Ther.

[CR30] Wang H, Shi J, Luo Y (2014). LIM and SH3 protein 1 induces TGFbeta-mediated epithelial-mesenchymal transition in human colorectal cancer by regulating S100A4 expression. Clin Cancer Res.

[CR31] Wang Y, Li Y, Shao P (2020). IL1beta inhibits differentiation of cementoblasts via microRNA-325-3p. J Cell Biochem.

[CR32] Zhang Z, Han Y, Sun G (2019). MicroRNA-325-3p inhibits cell proliferation and induces apoptosis in hepatitis B virus-related hepatocellular carcinoma by down-regulation of aquaporin 5. Cell Mol Biol Lett.

[CR33] Zhang Y, Ma LN, Xie Y (2020). MiRNA-802 inhibits the metastasis of colorectal cancer by targeting FOXE1. Eur Rev Med Pharmacol Sci.

[CR34] Zhao H, Zhang J, Shao H (2017). miRNA-340 inhibits osteoclast differentiation via repression of MITF. Biosci Rep.

